# Strategies for combating avian influenza in the Asia–Pacific

**DOI:** 10.5365/wpsar.2018.9.5.007

**Published:** 2018-12-18

**Authors:** Lisa Peters, Carolyn Greene, Eduardo Azziz-Baumgartner, Suizan Zhou, Socorro Lupisan, Wang Dayan, Aspen Hammond, Filip Claes, Elizabeth Mumford, Erica Dueger

**Affiliations:** a WHO Regional Office for the Western Pacific.; b United States Centers for Disease Control and Prevention.; c Research Institute for Tropical Medicine, Philippine Department of Health.; d National Institute for Viral Disease Control and Prevention, Chinese Center for Disease Control and Prevention.; e World Health Organization.; f Food and Agriculture Organization of the United Nations.; * This work was conducted on behalf of the WHO Health Emergencies Programme of the WHO Regional Office for the Western Pacific.

Avian, swine and other zoonotic influenza viruses may cause disease with significant impact in both human and animal populations. The Asia Pacific Strategy for Emerging Diseases (APSED), long recognizing the increased global impact of zoonotic diseases on human populations, has been used as the foundation for improving national preparedness and regional coordination for response to zoonotic diseases in the World Health Organization (WHO) Western Pacific Region. (*1*) APSED encourages multisectoral coordination at the human–animal–environment interface as the primary action required for zoonotic disease control. (*2*) In this article we emphasize the effectiveness of these multisectoral collaborations in responding to zoonotic diseases at the regional and country level, using avian influenza as an example.

In the 2006 version of APSED, the proposed approach for addressing zoonoses was: to strengthen regional mechanisms to support national-level collaborations between the animal, human and environmental health sectors; and to strengthen national-level capacity for collaboration between the animal and human health sectors. (*3*) The regional component was achieved through a tripartite collaboration of the Food and Agriculture Organization of the United Nations (FAO), World Organization for Animal Health (OIE) and WHO, which formalized a commitment to coordinate activities and risk reduction strategies at the human–animal–environment interface, taking a One Health approach in 2010. (*4*, *5*) The national component was addressed by developing national-level guidelines for establishing collaborations between national human and animal health sectors, providing a step-by-step approach to improve coordination of surveillance, information sharing, response and risk reduction. (*3*)

During the last five years, the emergence and spread of the H7N9 virus in domestic poultry and the occurrence of human cases in China have illustrated the importance of working at the human–animal–environment interface at the country and regional level. When the first human case of H7N9 virus infection was reported from China in March 2013, pandemic preparedness capacities were quickly tested. First, a swift, multisectoral response was undertaken by the Chinese Government to facilitate early detection and reporting of H7N9 in poultry and humans. (*6*) Then, the Chinese National Influenza Center shared H7N9 sequences, diagnostic test protocols and viruses with the Global Initiative on Sharing All Influenza Data (GISAID) public database, (*7*) the WHO influenza collaborating centres and the National Avian Influenza Reference Laboratory in Haerbin. These actions contributed greatly to the global risk assessment and response, including the selection and development of candidate human H7N9 vaccine viruses, vaccine potency and diagnostic reagents, as well as a better understanding of antigenicity, pathogenicity and transmissibility of the virus. (*8*) The Chinese Government also issued prevention and control guidelines including enhanced surveillance for influenza-like illness and severe acute respiratory infection in humans, improved case investigation and contact tracing and early treatment of human illness. (*9*) Meanwhile, at the regional level, multisectoral mechanisms were also activated that included increased surveillance in humans and poultry populations at border areas in Viet Nam, the Lao People's Democratic Republic and Myanmar and the sharing of information from China within the region.

Prior to 2017, only the low pathogenic avian influenza (LPAI) form of the H7N9 virus had been detected in poultry in China, with intermittent human cases, usually associated with poultry exposure. LPAI shows little to no clinical signs of infection in poultry, but it is important to monitor and control to prevent the spread to humans. In early 2017, H7N9 viruses that were highly pathogenic avian influenza (HPAI) were detected in poultry and humans in China. (*6*) Among the 759 human infections of H7N9 identified in China from October 2016 to September 2017, 27 were associated with HPAI H7N9. (*10*) HPAI often causes illness and death in poultry, facilitating strict control measures to stop the spread of disease among animals and to humans. Responses to this included the promotion of large-scale poultry farming by the Chinese Government (as opposed to higher-risk household or small-holder poultry holdings), centralized slaughtering, improved poultry product cold chain transportation and storage and expanded implementation of the “1110” strategy in live poultry markets. The 1110 strategy involves *1* daily cleaning, *1* weekly disinfection, *1* day of market closure every month and *0* live poultry stock overnight. (*11*) In September 2017, the Chinese national poultry vaccination programme with bivalent H5/H7 vaccine was launched. (*11*) In addition to targeted human and animal surveillance and control efforts, regular tripartite risk assessments based on updated national data have informed H7N9 response efforts. These response and control efforts were directly in line with the APSED goal of strengthening coordination at the human–animal interface and underscore the importance of continued regional improvements in this area.

The need for coordinated multisectoral preparedness to respond to acute zoonotic threats was also underscored in April 2017 when the Philippines detected its first outbreak of avian influenza in poultry. Rapid response teams were dispatched and samples were sent to an FAO reference centre laboratory. When avian influenza A(H5N6) was confirmed, the rapid response teams established a 1 km quarantine area and a 7 km control area around infected poultry farms. Strict animal surveillance and movement control measures were implemented and over 500 000 birds were destroyed. Concurrently, intensive surveillance was initiated at both hospital and community levels as well as community awareness campaigns. The acute H5N6 outbreak was resolved in poultry by September 2017 with no human cases detected, highlighting the importance of prioritizing multisectoral collaborations and preparedness efforts, even in countries that have not previously experienced major avian influenza outbreaks.

To continue to support national collaborations between animal, human and environmental health sectors, the WHO, OIE and FAO tripartite has been updating and expanding the tripartite zoonoses guide, entitled: Taking a Multisectoral, One Health Approach: A Tripartite Guide to Addressing Zoonotic Diseases in Countries. (*12*) The guide addresses coordinating mechanisms, planning and preparedness, surveillance and information sharing, coordinated investigation and response, joint risk assessments, risk communication, community engagement, joint risk reduction strategies and training and workforce development. The Joint Risk Assessment tool, (*13*) designed to evaluate risks and guide appropriate preparedness and response actions and risk communication, is included in the guide. The tool outlines the multisectoral organizational and technical processes and steps needed to assess the level of risk based on the likelihood and potential impact of zoonotic events. The Joint Risk Assessment tool (*13*) was designed to guide appropriate preparedness and response actions for zoonotic influenza;, however, it is equally applicable to other emerging zoonotic disease threats.

Successful country response efforts to avian influenza A(H7N9) in China and influenza A(H5N6) in the Philippines exemplify the importance of strong multisectoral collaboration for zoonotic diseases at both national and regional levels. The Asia Pacific Strategy for Emerging Diseases and Public Health Emergencies (APSED III) and the tripartite zoonoses guide will continue to assist countries in Asia and the Pacific to maintain and improve coordination between the human, animal and environmental health sectors for rapid and effective response efforts to emergent zoonotic influenza viruses.
